# The extent of shifts in vegetation phenology between rural and urban areas within a human‐dominated region

**DOI:** 10.1002/ece3.1990

**Published:** 2016-02-24

**Authors:** Martin Dallimer, Zhiyao Tang, Kevin J. Gaston, Zoe G. Davies

**Affiliations:** ^1^ Sustainability Research InstituteSchool of Earth and EnvironmentUniversity of LeedsWoodhouse LaneLeedsLS2 9JTUK; ^2^ Department of EcologyCollege of Urban and Environmental SciencesPeking UniversityBeijing100871China; ^3^ Environment and Sustainability InstituteUniversity of ExeterPenrynCornwallTR10 9EZUK; ^4^ Durrell Institute of Conservation and Ecology (DICE)School of Anthropology and ConservationUniversity of KentCanterbury, KentCT2 7NRUK

**Keywords:** Enhanced Vegetation Index, green infrastructure, greenspace, growing season, urban ecology, urban heat island, urbanization

## Abstract

Urbanization is one of the major environmental challenges facing the world today. One of its particularly pressing effects is alterations to local and regional climate through, for example, the Urban Heat Island. Such changes in conditions are likely to have an impact on the phenology of urban vegetation, which will have knock‐on implications for the role that urban green infrastructure can play in delivering multiple ecosystem services. Here, in a human‐dominated region, we undertake an explicit comparison of vegetation phenology between urban and rural zones. Using satellite‐derived MODIS‐EVI data from the first decade of the 20th century, we extract metrics of vegetation phenology (date of start of growing season, date of end of growing season, and length of season) for Britain's 15 largest cities and their rural surrounds. On average, urban areas experienced a growing season 8.8 days longer than surrounding rural zones. As would be expected, there was a significant decline in growing season length with latitude (by 3.4 and 2.4 days/degree latitude in rural and urban areas respectively). Although there is considerable variability in how phenology in urban and rural areas differs across our study cities, we found no evidence that built urban form influences the start, end, or length of the growing season. However, the difference in the length of the growing season between rural and urban areas was significantly negatively associated with the mean disposable household income for a city. Vegetation in urban areas deliver many ecosystem services such as temperature mitigation, pollution removal, carbon uptake and storage, the provision of amenity value for humans and habitat for biodiversity. Given the rapid pace of urbanization and ongoing climate change, understanding how vegetation phenology will alter in the future is important if we wish to be able to manage urban greenspaces effectively.

## Introduction

Urbanization and climate change are two of the major environmental challenges facing the world today. Determining the broad swathe of consequences of climate change on species, communities and ecosystems have been the leading focus of research in recent decades (e.g., Bunn 2009). One of the most well‐documented impacts has been the extension of the growing season for plants in temperate regions (e.g., Menzel and Fabian 1999; Noormets 2009). During the latter half of the 20th century, at mid‐ and high latitudes, a shift towards an earlier onset of spring and later autumn has been widely observed (Steltzer and Post 2009 and references therein). The magnitude of the phenomenon has been shown to be closely associated with changes in mean temperature (White et al. 1997; Fitter and Fitter 2002; Penuelas et al. 2002; White and Nemani 2003; Badeck et al. 2004; Chuine et al. 2004; Piao et al. 2006; Jeong et al. 2011; Cong et al. 2013). However, other factors such as water availability, precipitation, photoperiod length, nitrogen deposition, and CO_2_ concentrations are also known to influence vegetation phenology (Badeck et al. 2004; Korner and Basler 2010; Jeong et al. 2011; Cong et al. 2013).

Globally, the pace of land conversion to urbanized areas is rapid (Seto et al. 2012), with over half of the human population now living in towns and cities (United Nations 2013). Through the conversion of natural land surfaces to the built form, such as buildings, roads, and other sealed surfaces, urbanization radically alters many aspects of an ecosystem, including water availability, species composition, and soil properties (Gaston et al. 2010). One outcome of urbanization is an alteration of local and regional climate via the Urban Heat Island (UHI) effect, modifications to wind flow and turbulence, as well as shifts in patterns of cloud formation and precipitation (for reviews Gaston et al. 2010; Seto and Shepherd 2009). Temperature increases associated with UHI can be as great as several degrees Celsius, even in mid‐ and high latitude cities (e.g., Kershaw et al. 2010). Given the link between phenology and temperature, we would therefore expect seasonal patterns of vegetation growth and die‐back to differ between urban centers and surrounding non‐built‐up areas. While temperature shifts associated with urbanization are undoubtedly important, ground‐based observations indicate that the influence of urbanization on various aspects of the growing season is mediated by species and community composition, soil moisture, and topology (Fisher et al. 2007; Gazal et al. 2008; Hwang et al. 2011; Jochner et al. 2012), all of which can be substantially altered by urbanization.

Shifts in vegetation phenology can have a profound impact on ecosystem function, altering water, carbon, and energy balances, and affecting interspecific interactions and productivity (Schwartz 1998; White et al. 1999; Menzel 2000; Parmesan and Yohe 2003). This is particularly pertinent in urban areas where vegetation is key to the delivery of many important ecosystem services, including temperature mitigation (Susca et al. 2011; Park et al. 2012; Myint et al. 2013), pollution reduction (Manes et al. 2012; Pugh et al. 2012), carbon storage (Davies et al. 2011), recreational opportunities for human residents (EEA 2009), and habitat for biodiversity (Chace and Walsh 2006; McKinney 2008; Dallimer et al. 2012). Indeed, green infrastructure is increasingly being recognized as a critical component of urban areas, improving the quality of the environment for inhabitants (Gaston 2010; Niemela et al. 2010; Keniger et al. 2013).

Here, we undertake an explicit comparison of vegetation phenology between urban and adjacent rural zones. Using satellite‐derived data, we examine variation in the growing season across the 15 largest cities in Britain. Our aim (cf. White et al. 2002) is to do this without assessing the longer‐term temporal trends associated with climate change. We therefore restrict our analyses to the first decade of the 21st century, as temperatures (Cane 2010; Wang et al. 2011) and phenological signals in vegetation (e.g., Jeong et al. 2011; Piao et al. 2011; Wu and Liu 2013) remained relatively static during this time.

We purposefully carry out our analyses in a human‐dominated region. Some 80% of the UK population already lives in towns and cities, a proportion that the rest of the world is predicted to approach by 2050 (United Nations 2013). Further, we include all land covers in our dataset. Much of the existing literature has compared urban areas with nearby forested/natural landscapes (e.g., White et al. 1997; Zhang et al. 2004; Wu and Liu 2013), thereby excluding, for example, agricultural land, where management influences phenology. Some urban–rural differences in growing season that have been ascribed to urbanization might, therefore, be confounded by the radically different type, scale and management of vegetation occurring in urban compared to forested/natural areas. Our study cities are surrounded by a mosaic of land covers, such as patches of woodland and shrub, grass and croplands, which, although different from land covers in urban areas, encompass many of the types of heterogeneous vegetation structure and cover (e.g., woodland, shrub patches, amenity grassland) that are typically found in British cities. We test the following statements: (i) the growing season will be longer in urban compared to neighboring rural areas across the major cities of Britain, and (ii) any declines in growing season length that are associated with latitude will be notably less in urban areas.

## Materials and Methods

### Data acquisition and processing

**Figure 1:**
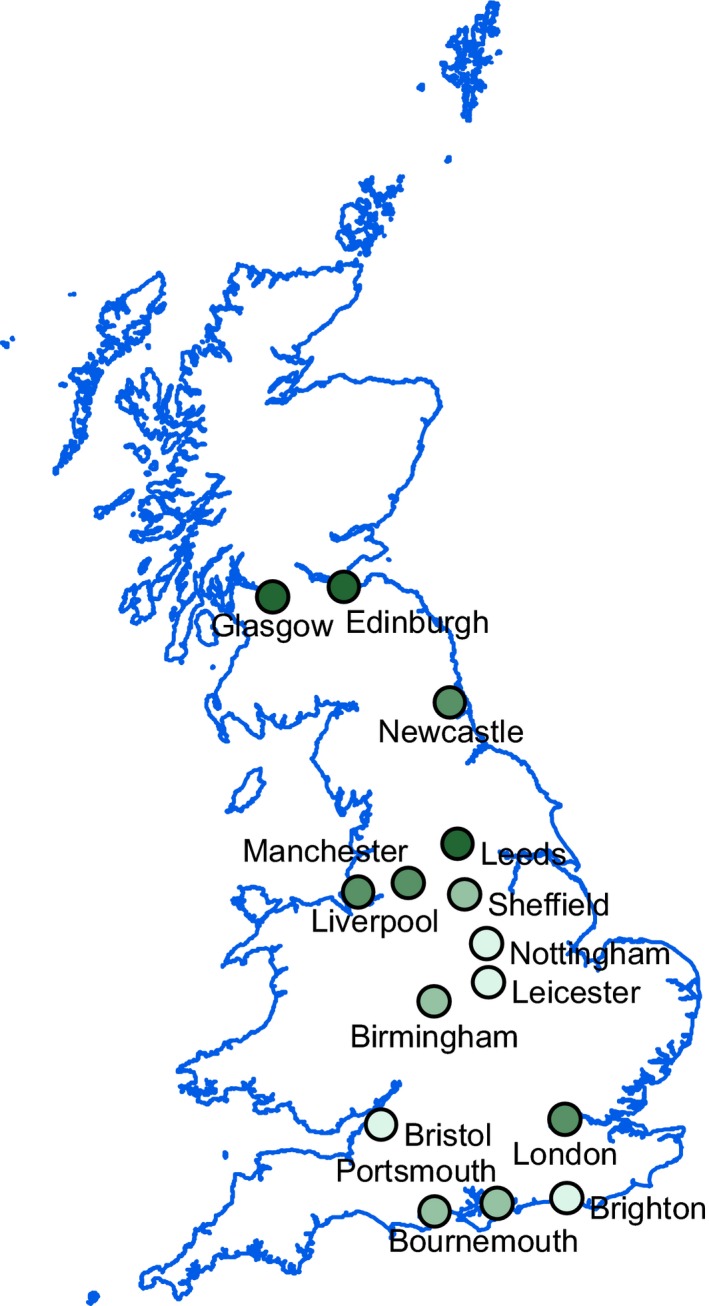
The location of the 15 study cities (Latitudes 50.72 to 55.95N) in Britain, northwest Europe. Darker green shading indicates increasing proportion of each city that was recorded as greenspace.

Satellite‐derived vegetation indices, related to the fraction of photosynthetically active radiation absorbed by plants, are widely used as a surrogate for vegetation activity (Huete et al. 2002; Sims et al. 2006; White et al. 2009). In this study, we use Moderate Resolution Imaging Spectroradiometer Enhanced Vegetation Index (MODIS‐EVI) data to examine phenological trends within Britain's 15 largest cities (Fig. 1; Appendix S1 and Table S1). The dataset has a spatial resolution of 250 × 250 m, a temporal resolution of 16 days (16‐day composite period (Justice et al. 2002), and we extracted data spanning a 9‐year period in the UK from February 2000 (when MODIS‐EVI data first became available) to December 2009. The MODIS‐EVI data were downloaded from the Global Land Cover Facility (http://glcf.umd.edu/).

**Figure 2:**
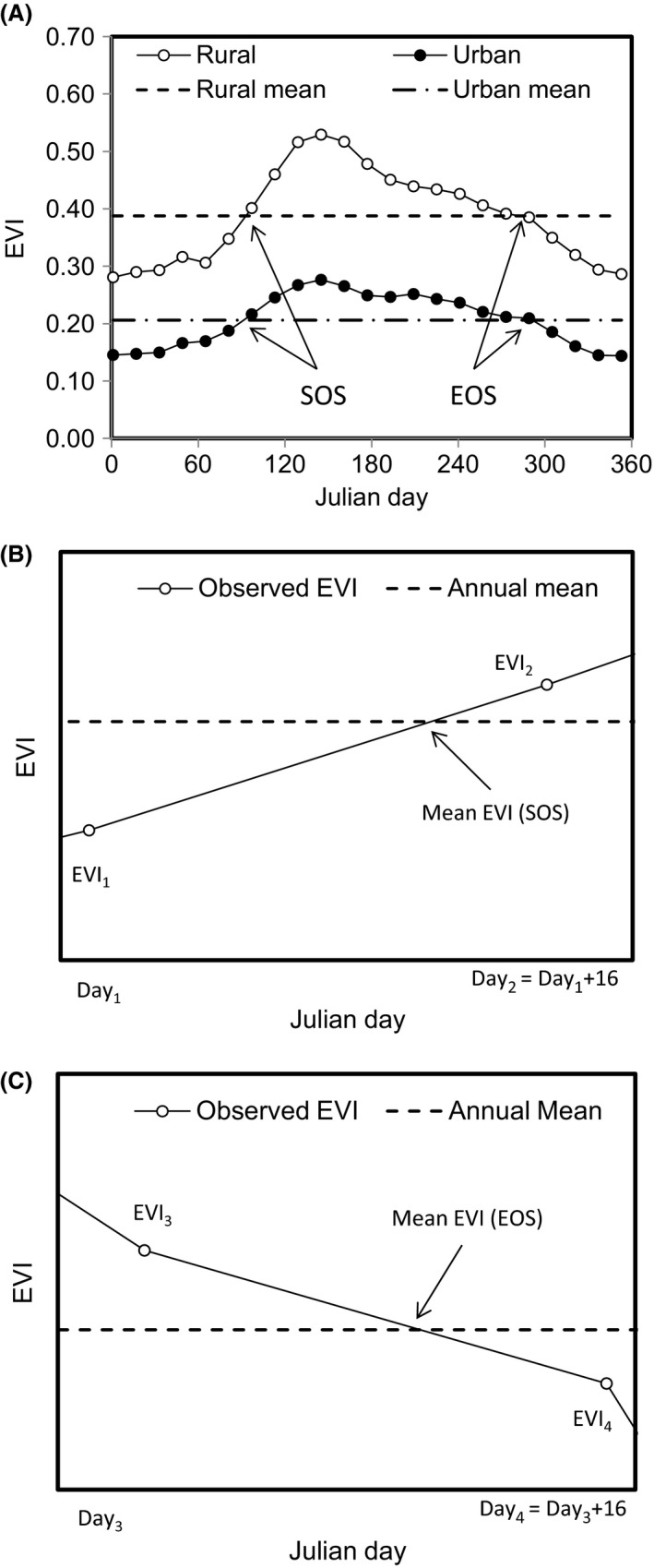
(A) An example of the annual pattern in biweekly Moderate Resolution Imaging Spectroradiometer Enhanced Vegetation Index (MODIS‐EVI) data, averaged across all years, for the urban extent of the city of Birmingham (closed circles; dashed line, annual mean EVI) and its surrounding rural zone (open circles; dotted line, annual mean EVI). SOS and EOS indicate the start and end of the growing season, respectively. To interpolate the estimated date of EOS and SOS from the 16‐day interval of the EVI data, we assumed that EVI increased in spring, and decreased in autumn linearly within the 16 days of the interval. Therefore, the estimated SOS date (when the mean EVI intersected with observed EVI) is calculated as follows: SOS = day_1_ + (mean EVI − EVI_1_)/(EVI_2_ − EVI_1_) × 16. Where day_1_ and day_2_ are the neighboring dates of the EVI values. Similarly, we calculated the EOS as following: EOS = day_3_ + (mean EVI − EVI_4_)/(EVI_3_ − EVI_4_) × 16. (B) and (C) illustrate in more detail how linear interpolation between biweekly data points was used.

In order to investigate how the growing season within cities might be altered as a result of urbanization, comparative baseline data were required for adjacent rural zones. The urban extent of each city was delimited according to the 2006 Ordnance Survey definition using a Geographic Information System (GIS). Surrounding rural zones were defined as all non‐built‐up land uses (determined using Landsat TM; Appendix S2) lying in a buffer between 2 and 5 km around the urban boundary. Based on these urban and rural zones, within the GIS, we calculated the biweekly mean EVI for each study city and its associated rural area by averaging across all EVI pixels contained within the zones. The start of the growing season (SOS) each year was defined as the first day that the EVI increased above the annual mean EVI in the spring. The end of the growing season (EOS) was taken as the day the EVI decreased below the annual mean EVI in the autumn (Zhou et al. 2001; Suzuki et al. 2003). Finally, the length of the growing season (LOS) was the difference between the SOS and EOS. As the proportion of vegetation cover within each city and its adjacent rural area differs, a specific annual mean EVI threshold was applied to each zone (Fig. 2). This approach is equivalent to, but simpler than, the phenology fitting curve used in other studies (Zhou et al. 2001; Suzuki et al. 2003).

In some years, peaks in EVI above the annual mean EVI threshold occurred during winter. Such peaks were characterized by a single period of high EVI followed by an immediate reduction and are likely to be due to factors such as cloud, atmosphere, and solar zenith angle. As it is not possible for plants in temperate regions to complete their growth phase in such a short period of time during the winter, we excluded EVI peaks from our definitions of SOS and EOS. We did this by discounting peaks in EVI that occurred prior to day 70 (11 March) or after day 315 (11 November); dates that were chosen based on our experience of the study region and inspecting the annual form of EVI. A further potential complication is the theoretical situation where low points in EVI could occur in the rural zone where that area is cultivated and harvested. Although low points did occur, EVI never dropped below the mean annual EVI prior to the autumn “green down.” We defined EVI thresholds using both mean and median EVI. There were no substantive differences in the outputs, so here we report results based on the mean only.

The choice of method used to determine the SOS and EOS can lead to considerable variation and limit the comparability of different studies. For example, satellite‐derived data tend to deliver SOS dates earlier and EOS later than ground‐based observations (White et al. 2009; Zhu et al. 2012), with heterogeneity in vegetation cover as one explanation for why this occurs (Badeck et al. 2004). Equally, the exact methodology used to estimate the SOS from the satellite data itself will lead to different dates being estimated. White et al. (2009) compared 10 methods for calculating the SOS, from empirical approaches to mathematical models. They found that no method could claim to be automatically superior to the others. Similarly, Cong et al. (2013) found that although there were significant differences between techniques in their estimates of the SOS and EOS, all were internally consistent and able to reveal the same patterns within the data. We therefore opted for a transparent and straightforward method for ascertaining the SOS and EOS (as outlined above). Finally, several methods can also be applied to interpolate between the EVI data points, such as linear, quadratic, cubic, and polynomial interpolation. The linear method might estimate an earlier SOS and later EOS, given a concave shape of EVI pattern, but a later SOS and earlier EOS, given a convex shape of EVI pattern (see Fig. 2 B and C). The selection of methods depends on the shape of the temporal changes of EVI, how many dates with EVI data are available, and the purpose of the phenological phase estimation. When comparing the temporal and spatial patterns of phenological phases, it is important to select a method that is comparable among different times and different sites. We therefore selected linear interpolation, which is not only simple and reliable, but compatible among different datasets and consistent with our desire to apply a straightforward and consistent method.

In this study, we wished to understand the impact of urbanization as a whole on vegetation phenology. This could be mediated through many possible drivers (e.g., temperature, water availability, nitrogen deposition, vegetation community composition, variation between native and non‐native plant species in responses to photoperiod and/or temperature). We therefore did not attempt to disentangle their relative importance and instead examined whether differences in the SOS, EOS, and LOS between each city and its neighboring rural zone were associated with some key characteristics of urban form (the proportion of greenspace, dwelling density, extent of the urban area, distance to the nearest major urban area, and disposable household income; Table S3). Following previous studies, we predicted that the difference in SOS, EOS, and LOS between each city and its neighboring rural zone would be negatively associated with the proportion of greenspace in a city (cf. temperature mitigation associated with vegetation in cities; Susca et al. 2011; Park et al. 2012; Myint et al. 2013). We anticipated a similar association with disposable income, given that socio‐demographics are often associated with many aspects of vegetation structure and coverage in cities (e.g., Hope et al. 2003; Luck et al. 2009). In contrast, we predicted that the difference in SOS, EOS, and LOS between each city and its neighboring rural zone would be positively associated with dwelling density (a metric of how intensively built‐up an urban area is) and the extent of the urban area. Finally, cities can raise temperatures in a broad swathe of rural land around them (Zhang et al. 2004; Elmore et al. 2012). Rural areas close to several large cities are therefore likely to experience higher temperatures than more isolated areas, reducing the phenological differences between a city and its surrounding rural zone. We therefore predicted that differences in phenology would be lower when major urban areas were closer together.

### Statistical analyses

Linear regression was used to explore temporal trends for each of the growing season characteristics in the study cities and their neighboring rural zones which might be attributable to large scale changes in, for example, climate. As no relationships between the SOS, EOS, or LOS and time were apparent, either at the individual city level or for all cities combined (Table S2), mean values for these variables across the 10 year period were used in all further analyses. Across all the cities, paired *t*‐tests were used to assess whether urbanization resulted in a significant difference in any of the growing season characteristics between urban and rural zones. At the individual city level, differences between urban areas and adjacent rural zones for the SOS, EOS, and LOS were investigated using Wilcoxon signed‐rank tests, as the data were not consistently normal.

To determine whether SOS, EOS, and LOS varied with latitude, linear regression was applied (Table S3). We hypothesized that, if latitudinal trends were present, they would be less pronounced (i.e., *β* lower) within the cities, and tested for this by including an interaction term between latitude and zone (rural or urban). If this term were to be significant, then this would be evidence that *β*s, and therefore latitudinal trends, differed between zones. Finally, we assessed the strength of any association between urban form and the difference in SOS, EOS, and LOS between urban and rural areas with partial Spearman's rank correlations, which allowed us to account for the likely influence of latitude. Our sample size (*N* = 15) precluded us from undertaking more complex multivariate analyses.

## Results

**Figure 3:**
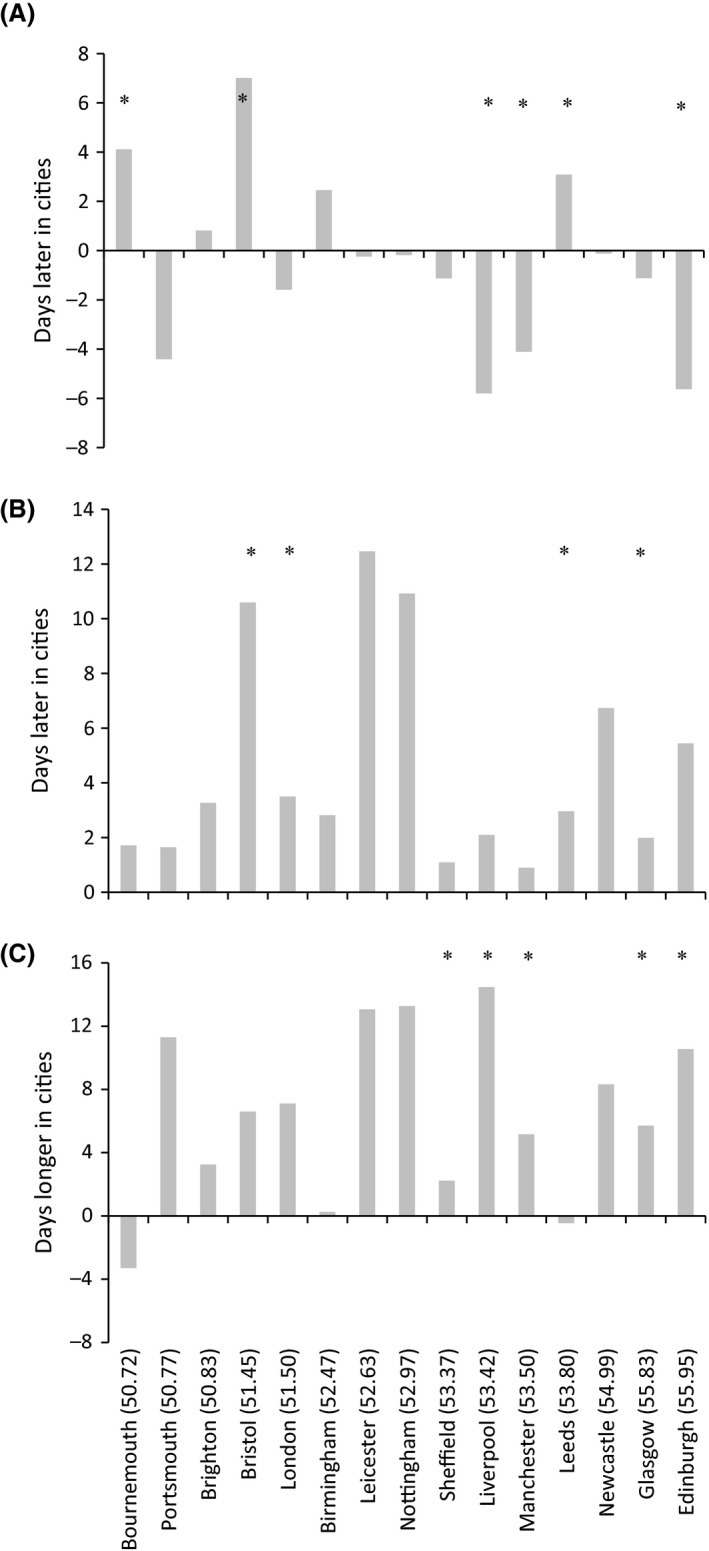
Differences in the (A) start of the growing season (SOS), (B) end of the growing season (EOS) and (C) length of growing season (LOS), for each study city (latitude in brackets) when compared to its surrounding rural area. * indicates a significant difference (*P* < 0.05) between the urban and rural zones (Table S4).

When considering individual cities, no uniform pattern emerged in relation to the SOS in urban versus rural zones (Fig. 3A; Table S4); the SOS was significantly earlier in three urban centers, compared to adjacent rural areas, but significantly delayed in another two. In contrast, the EOS was consistently later in all urban areas, significantly so for four cities (Fig. 3B; Table S4). The LOS was extended in 13 cities, five of which were significant (Fig. 3C; Table S4). In all cases, differences were modest compared to the temporal resolution of the MODIS data.

Combining the data from across all 15 British cities, the LOS was significantly longer in urban versus rural areas by 8.8 days (192 days and 183 days, respectively). The mean SOS occurred on 3 April in urban areas compared to 4 April in adjacent rural zones, due to an average, non‐significant, advance of 0.8 days (Table 1). Similarly, on average, the EOS was 8.0 days later in urban areas than rural zones, with a mean date of 12 October as opposed to 4 October.

**Table 1:** Paired *t*‐tests assessing differences in the growing season characteristics between urban and rural zones across 15 British study cities: start of season, SOS; end of season, EOS; length of season, LOS

Growing season variable	Zone	Mean	SE	df	*t*	*P*
SOS	Urban	93.36	1.23	24.721	0.376	0.710
	Rural	94.18	1.80			
EOS	Urban	285.25	1.87	24.336	−2.357	0.027
	Rural	277.30	2.81			
LOS	Urban	191.88	1.89	27.448	−3.036	0.005
	Rural	183.12	2.18			

**Figure 4:**
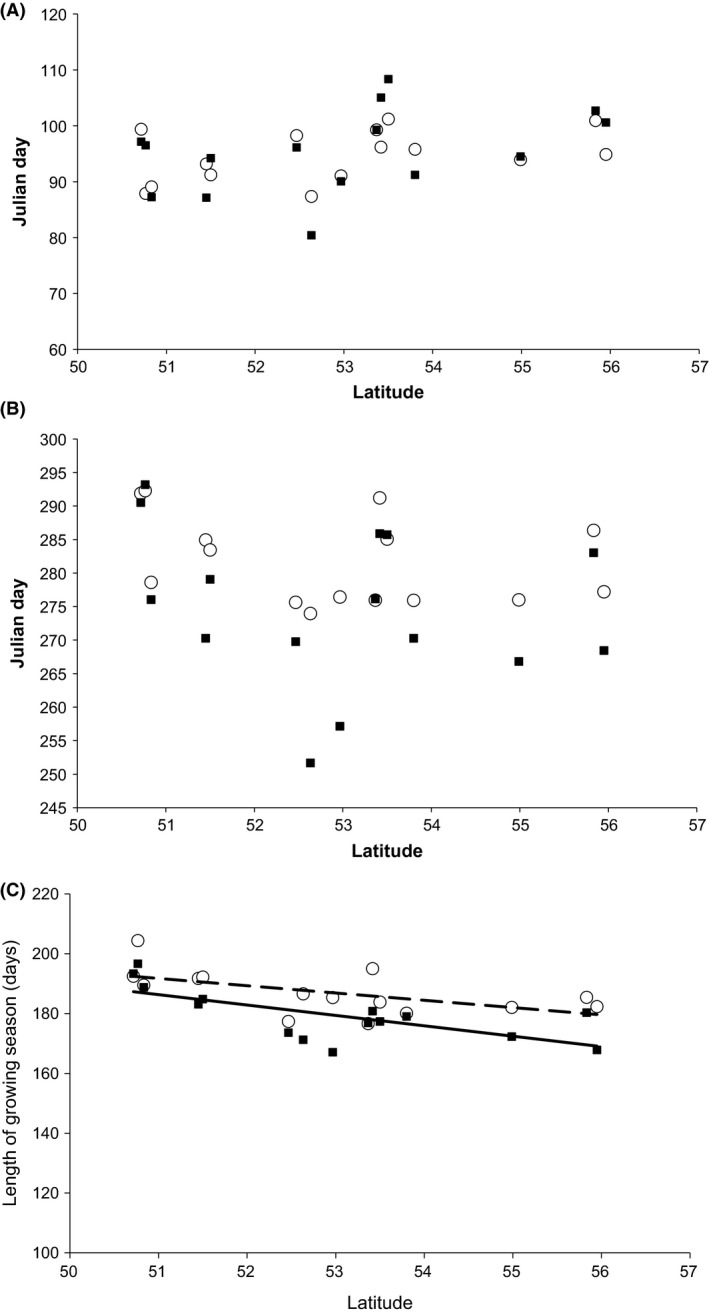
The relationship between latitude and the (A) start of the growing season (SOS), (B) end of the growing season (EOS), and (C) length of growing season (LOS), for each of the 15 British study cities (open circles) and their surrounding rural areas (closed squares). Lines (dashed, cities; solid, rural areas) indicate a significant relationship (*P* < 0.05) with latitude.

Although the SOS was delayed with increasing latitude (1.9 and 0.9 days per degree of latitude in rural and urban areas, respectively), these trends were not significant (Fig. 4A; Table 3). Similarly, there was a non‐significant advance in EOS, of 1.5 days in both rural and urban areas with each degree of latitude (Fig. 4B; Table 3). However, the LOS decreased significantly, shortening by 3.4 days and 2.4 days per degree of latitude in rural and urban zones respectively (Fig. 4C; Table 3). Across Britain, from south to north, this equates to a 17.6‐day reduction in the LOS in the rural zones surrounding the study cities, and a 12.5‐day decline in LOS within urban areas (Fig. 4C). For all three growing season characteristics, the latitudinal trends were weaker (but not significantly so) in urban than rural areas, but interaction terms, and therefore, the differences between the slopes were not significant (Table 2).

**Table 2:** Linear regression models of the relationships between each growing season characteristics and latitude, for the urban and rural zones associated with Britain's largest 15 cities: start of season, SOS; end of season, EOS; length of season, LOS

Growing season variable	Zone	*β* a	SE	*P*
SOS (*R* ^2^ = 0.13)	Urban	0.94	0.72	0.213
	Rural	1.89	0.99	0.078
	Latitude × Zone Interaction^b^	−0.95	1.22	0.443
EOS (*R* ^2^ = 0.14)	Urban	−1.46	1.09	0.203
	Rural	−1.48	1.70	0.399
	Latitude × Zone Interaction^b^	0.02	2.06	0.991
LOS (*R* ^2^ = 0.50)	Urban	−2.39	0.97	0.028
	Rural	−3.37	0.98	0.005
	Latitude × Zone Interaction^b^	0.97	1.38	0.487

a General form of the regression equation *y* = *α *+ *β* 1 (Latitude) + *β* 2 (Zone) + *β* 3 (Latitude × Zone), where zone is a dummy with the value 0 for rural and 1 for urban. Intercept (*α*) is not reported. *β* represent the slope of the relationship between the growing season characteristic and latitude. The interaction terms are the difference between those slopes.

b If significant, this interaction term would indicate that latitudinal trends differed between urban and rural zones.

**Table 3:** Partial Spearman's rank correlations between the advance/delay (days) in each growing season characteristic in urban areas, compared to rural zones and key aspects of, while accounting for the likely influence of latitude across the 15 study cities: start of season, SOS; end of season, EOS; length of season, LOS

Growing season variable	Greenspace (%)	Dwelling density (no./ha)	Urban extent (ha)	Distance to nearest major city (km)	Household disposable income (GB£)
SOS	−0.08	−0.40	0.01	−0.03	0.18
EOS	−0.39	0.02	−0.03	−0.23	−0.48
LOS	−0.49	0.35	−0.23	−0.31	−0.71**

Significance levels (*P < 0.05; **P < 0.01) adjusted to correct for multiple tests using the Holm–Bonferroni method.

Shifts in the growing season characteristics were modest and, after accounting for the influence of latitude in a partial correlation, were not significantly associated with the aspects of built urban form examined (percentage of greenspace, dwelling density, extent of the urban area, distance to nearest major urban area). However, LOS was negatively associated with disposable household include (Table 3).

## Discussion

Across Britain's 15 largest cities, urbanization has extended the growing season by an average of 8.8 days within urban areas, compared to rural surroundings, by prolonging the end of the season (Table 1) (cf. Elmore et al. 2012; Garonna et al. 2014). However, this figure masks considerable variation at an individual city level, with the differences in the length of the growing season ranging between 3.3 days shorter in the urban versus rural areas of Bournemouth, compared to 13.1 days longer for Leicester (Fig. 3C). Much of the variability can be explained by the geographic location of the cities (Table 1), with the growing season estimated as being 17.6 and 12.5 days longer in rural and urban areas, respectively, for the lowest compared to the highest latitude (Fig. 4C). Although the modest compared to the relatively coarse temporal resolution of MODIS data, as long as a consistent methodology is used, vegetation phenology variables estimated from satellite data are robust (Cong et al. 2013), we can thus conclude that trends detected in our study are sound.

Although we detected no uniform pattern for the start of the growing season in urban areas to be earlier than rural zones, the end of the growing season was always delayed. This suggests that the length of the season in urban zones might be more strongly determined by autumn, rather than spring, vegetation phenology. The precise timing of the end of the growing season is inherently more difficult to measure than the start due to the gradual “green down” observed in temperate areas (cf. a rapid “green‐up” at the onset of warmer temperatures in the spring), which perhaps is part of the reason why the importance of changes in autumn phenology in driving growing season length remains understudied (Garonna et al. 2014). Nevertheless, our results reflect those for Europe as a whole (Garonna et al. 2014) as well as those along gradients of urbanization in mid‐Atlantic forests in North America (Elmore et al. 2012). Autumn phenology is strongly influenced by soil moisture and hydrology (Garonna et al. 2014; Yang et al. 2015), so the patterns we observed might be at least partly driven by changes to these that occurs in cities (Elmore et al. 2012). Differences in species composition between urban and rural zones might also be important (Elmore et al. 2012).

Climate change is thought to be the major driver in the advance and extension of growing seasons in temperate latitudes (e.g., Schwartz 1998; Menzel and Fabian 1999). Nonetheless, ambient climate can also be altered by land‐use changes associated with urbanization (e.g., Imhoff et al. 2010; Li et al. 2011). For instance, Kershaw et al. (2010) modeled UHI for the largest cities in the UK and concluded that average spring temperatures were between 0.2 and 1.9°C warmer (for Leicester and Newcastle, respectively) than the surrounding rural landscape. One explanation for the variation in UHI is the differences in urban form (e.g., percentage of greenspace, dwelling density, extent of the urban area) between cities. We might, therefore, also expect variation in city‐wide attributes of urban form to be associated with vegetation phenology. However, after accounting for latitude, we did not uncover any significant associations. Including measures of the UHI for each city in our analyses might have increased our understanding of the intracity variability, but our overall finding is consistent with that of Zhang et al. (2004), who also failed to detect a relationship between phenological differences between adjacent urban and rural zones and city size.

The vegetation phenological variability observed our study at an individual city level is likely to be attributable to local‐scale vegetation and topographic features that characterize each urban area and directly influence the extent of the UHI effect experienced. For example, the specific characteristics of buildings can alter temperature patterns in urban landscapes (Myint et al. 2013), while trees and shrubs mitigate UHI (Susca et al. 2011; Park et al. 2012; Myint et al. 2013). Even small areas of vegetation can reduce UHI effects in their immediate vicinity (Oliveira et al. 2011), as can water bodies, such as rivers (Hathway and Sharples 2012). Furthermore, vegetation phenology can be modified by many factors, including disease, competition, soil condition, nutrient and water availability, and weather patterns (Menzel 2000). Similarly, the composition of vegetation communities differs between rural and urban zones, and across the latitudinal range of our study. Given that the responses of individual species to climate change are enormously varied (for a summary, see Korner and Basler 2010), this might confound our ability to uncover consistent phenological patterns with remote sensed data.

Interestingly, a smaller difference in LOS was associated with higher city‐scale mean disposable household income. In the desert city of Phoenix, USA, higher neighborhood household incomes were associated with cooler temperatures (Jenerette et al. 2011). If this relationship was also apparent in our region, this would translate to a smaller difference in vegetation phenology between urban and rural settings. Indeed, income and socio‐economic status is often associated with vegetation structure, type, species richness, and community composition in urban areas (e.g., Hope et al. 2003; Luck et al. 2009). This therefore provides a plausible mechanism through which this association could be accounted for.

The spatial extent over which cities alter local climate can extend beyond the urban boundary. For example, in eastern North America, the climate influence of cities extended up to 10 km into the adjacent rural zone (Zhang et al. 2004). Similarly, Elmore et al. (2012) found that the influence of urban land use could be detected up to 32 km from large cities. Differences in vegetation phenology between urban and rural zones are therefore likely to be mediated by the extent to which rural areas are within a “climate shadow” of other nearby cities. Rural areas close to many cities might experience elevated temperatures compared to more isolated zones. In our study, differences in phenology between urban and rural zones were not associated with the distance between large cities. One explanation for the lack of an association could be that in our study region, the high human population density in Britain means that the majority of the rural landscape is impacted by urban “climate shadows” to some extent, and future work could focus on the size and intensity of these effects. In addition, we deliberately carried out our analyses in a human‐dominated region and included all land covers in our dataset. Much of the existing literature has compared urban areas with nearby forested/natural landscapes (e.g., White et al. 1997; Zhang et al. 2004; Wu and Liu 2013). Some urban–rural differences in growing season that have been ascribed to urbanization might, therefore, be confounded by the different type, scale and management of vegetation occurring in urban compared to forested/natural areas. In our region, the study cities are surrounded by a mosaic of land covers, such as patches of woodland, scrub, grasslands, and arable crop, which, although different from land covers in urban areas, encompass many of the types of heterogeneous vegetation structure and cover (e.g., woodland, scrub patches, amenity grassland; Davies et al. 2011) that are typically found in British cities.

## Limitations and Conclusions

Solely relying on satellite‐derived measures of phenology is likely to have restricted our ability to detect consistent signals for how urbanization might impact the growing season. For example, EVI response curves for rural areas were often characterized by a peak in growth in May–June, followed by a decline in July and a lower plateau in August–September. This is likely to reflect that large areas are covered by agricultural crops and grasses that ripen and are harvested/cut for silage in July–August. This pattern is likely to be less apparent in urban areas where frequent mowing and irrigation might keep vegetation greener for longer, leading to an apparent delay in the end of the growing season. In contrast, water shortages for street trees and pollution might both act in the opposite direction. In spring, increased temperatures in urban areas will generally mean that plants start growing earlier than they otherwise would. However, vegetation composition in urban areas is often very different from rural landscapes (e.g., see Dallimer et al. 2012) and urban vegetation can include many non‐native species whose phenology might be more temperature sensitive than native species, increasing the chances of observing an earlier start to the growing season. Disentangling the relative importance of these effects, among others, is extremely challenging and a topic for future research.

Vegetation is a key component of urban areas, delivering many ecosystem services such as temperature mitigation, pollution removal, carbon uptake, and storage, the provision of amenity value for humans and habitat for biodiversity. Given the rapid pace of urbanization (United Nations 2013) and ongoing climate change (IPCC 2013), understanding how vegetation phenology will alter in the future is important if we wish to be able to manage urban greenspaces effectively. The impacts of an extended growing season on vegetation communities are likely to be complex, not least because individual species and functional groups will respond differently (Korner and Basler 2010; Liang et al. 2011), with factors such as winter chilling, photoperiod, and temperature constraining them in varied and interacting ways. Nonetheless, the presence of “green” for longer in temperate urban areas could act to reduce local temperatures, not only through direct transpiration effects and shading (thereby reducing the UHI (e.g., Schwartz 1996)), but also indirectly by mitigating warming via carbon storage and sequestration (Penuelas et al. 2009). A longer growing season might also be beneficial for urban and peri‐urban agriculture and food production, especially as the influence of a city on climate can extend outside the built‐up area.

## Conflict of Interest

None declared.

## Supporting information


**Appendix S1.** Selection of study cities.
**Appendix S2.** Determining non‐developed land‐uses within the surrounding rural zones.
**Table S1.** Characteristics of Britain's largest ‘Urban Areas', in descending order of size, from the Office for National Statistics (ONS 2005).
**Table S2.** Linear regression models used to explore changes in the start of the growing season (SOS), end of the growing season (EOS), and length of the growing season (LOS), between 2000 and 2009, in Britain's 15 largest cities (listed in order of ascending latitude).
**Table S3.** Location and urban form characteristics Britain's 15 largest cities (listed in order of ascending latitude).
**Table S4.** For each individual city (listed in order of ascending latitude), vegetation growing season median start and end Julian days, and length of growing season (SOS, EOS and LOS respectively).
