# Supplementary material for: The extent of shifts in vegetation phenology between rural and urban areas within a human‐dominated region
Source: Ecol Evol. 2016 Feb 24;6(7):1942–53. doi: 10.1002/ece3.1990 (PMC4831430; doi:10.1002/ece3.1990)
Supplement: Supplementary file 1 — Appendix S1. Selection of study cities. Appendix S2. Determining non‐developed land‐uses within the surrounding rural zones. Table S1. Characteristics of Britain's largest ‘Urban Areas', in descending order of size, from the Office for National Statistics (ONS 2005). Table S2. Linear regression models used to explore changes in the start of the growing season (SOS), end of the growing season (EOS), and length of the growing season (LOS), between 2000 and 2009, in Britain's 15 largest cities (listed in order of ascending latitude). Table S3. Location and urban form characteristics Britain's 15 largest cities (listed in order of ascending latitude). Table S4. For each individual city (listed in order of ascending latitude), vegetation growing season median start and end Julian days, and length of growing season (SOS, EOS and LOS respectively). [file ECE3-6-1942-s001.docx]

**Supporting Information**

**Appendix 1: Selection of study cities**

The 15 study cities used for our analyses were selected on the basis of their associated ‘Urban Area’ human population size, as recorded during the 2001 census (Table S1; ONS 2005; Dallimer et al. 2011).

**Appendix 2: Determining non-developed land-uses within the surrounding rural zones**

Landsat Thematic Mapper (TM) images, comprising six spectral bands at a spatial resolution of 30 x 30 m, were used to explore rural land-use. TM data were acquired for spring/summer (April–September) 2006. Eight image scenes were needed to cover the 15 cities in each year. The Landsat TM data were downloaded from the Global Land Cover Facility (GLCF; <http://glcf.umiacs.umd.edu/index.shtml>) and interpreted using the image processing software ERDAS IMAGINE 8.4 (ERDAS Inc). The image scenes for each year were combined, prior to overlaying a 2-5 km buffer around the urban extent of each of the 15 cities and extracting the relevant data. The maximum likelihood method, based on the six spectral bands, was then used to classify the images into two land-use types: developed and non-developed.

**Table S1:** Characteristics of Britain’s largest ‘Urban Areas’, in descending order of size, from the Office for National Statistics (ONS 2005).

| **Urban area** | **City** | **Population** | **Area (km^2^)** | **Population density (people/km^2^)** |
| --- | --- | --- | --- | --- |
| Greater London | London | 8,278,251 | 1,623.37 | 5,099 |
| West Midlands | Birmingham | 2,284,093 | 599.72 | 3,809 |
| Greater Manchester | Manchester | 2,240,230 | 556.72 | 4,024 |
| Greater Glasgow | Glasgow | 1,168,270 | 368.47 | 3,171 |
| West Yorkshire | Leeds | 1,499,465 | 370.02 | 4,052 |
| Tyneside | Newcastle | 879,996 | 210.91 | 4,172 |
| Liverpool | Liverpool | 816,216 | 186.17 | 4,384 |
| Nottingham | Nottingham | 666,358 | 158.52 | 4,204 |
| Sheffield | Sheffield | 640,720 | 162.24 | 3,949 |
| Bristol | Bristol | 551,066 | 139.78 | 3,942 |
| Brighton/Worthing/Littlehampton | Brighton | 461,181 | 94.09 | 4,902 |
| Edinburgh | Edinburgh | 452,194 | 120.11 | 3,765 |
| Portsmouth | Portsmouth | 442,252 | 94.52 | 4,679 |
| Leicester | Leicester | 441,213 | 101.64 | 4,341 |
| Bournemouth | Bournemouth | 383,713 | 108.15 | 3,548 |

**Table S2:** Linear regression models used to explore changes in the start of the growing season (SOS), end of the growing season (EOS), and length of the growing season (LOS), between 2000 and 2009, in Britain’s 15 largest cities (listed in order of ascending latitude).

| **City** | **Zone** | **SOS** | | | **EOS** | | | **LOS** | | |
| --- | --- | --- | --- | --- | --- | --- | --- | --- | --- | --- |
|  |  | **β** | **S.E.** | **p** | **β** | **S.E.** | **p** | **β** | **S.E.** | **p** |
| Bournemouth | Rural | -1.03 | 1.13 | 0.389 | -1.62 | 2.60 | 0.550 | -0.59 | 2.63 | 0.828 |
|  | Urban | -1.41 | 1.32 | 0.317 | -2.00 | 2.59 | 0.462 | -0.59 | 2.90 | 0.844 |
| Portsmouth | Rural | -0.97 | 1.00 | 0.356 | 0.06 | 3.02 | 0.986 | 1.03 | 2.78 | 0.721 |
|  | Urban | 2.02 | 1.53 | 0.224 | -0.34 | 3.04 | 0.913 | -2.36 | 2.87 | 0.435 |
| Brighton | Rural | 1.25 | 1.04 | 0.262 | -3.81 | 4.19 | 0.391 | -5.05 | 3.74 | 0.213 |
|  | Urban | 1.34 | 1.18 | 0.286 | -3.53 | 3.67 | 0.363 | -4.88 | 3.24 | 0.171 |
| Bristol | Rural | 0.14 | 1.27 | 0.916 | -3.52 | 3.24 | 0.309 | -3.65 | 2.33 | 0.155 |
|  | Urban | 0.50 | 1.49 | 0.746 | -1.52 | 1.73 | 0.405 | -2.02 | 2.24 | 0.392 |
| London | Rural | -0.09 | 1.18 | 0.943 | -2.40 | 3.29 | 0.487 | -2.31 | 3.02 | 0.466 |
|  | Urban | -0.08 | 0.82 | 0.924 | -1.31 | 2.32 | 0.589 | -1.23 | 2.09 | 0.574 |
| Birmingham | Rural | -0.44 | 1.14 | 0.713 | -3.90 | 3.74 | 0.327 | -3.46 | 3.87 | 0.397 |
|  | Urban | -0.53 | 1.14 | 0.656 | -0.72 | 2.41 | 0.773 | -0.19 | 2.89 | 0.950 |
| Leicester | Rural | -0.32 | 0.88 | 0.726 | 0.70 | 4.80 | 0.888 | 1.01 | 4.57 | 0.829 |
|  | Urban | -0.67 | 1.25 | 0.603 | -1.02 | 2.88 | 0.733 | -0.34 | 2.71 | 0.902 |
| Nottingham | Rural | -1.37 | 1.43 | 0.366 | 3.70 | 3.78 | 0.356 | 5.06 | 4.02 | 0.243 |
|  | Urban | -0.95 | 1.73 | 0.598 | 0.15 | 2.37 | 0.950 | 1.10 | 2.43 | 0.662 |
| Sheffield | Rural | -1.53 | 1.03 | 0.176 | -0.10 | 2.64 | 0.972 | 1.43 | 2.94 | 0.638 |
|  | Urban | -0.96 | 1.00 | 0.365 | 1.94 | 1.45 | 0.220 | 2.90 | 1.86 | 0.158 |
| Liverpool | Rural | -0.31 | 0.90 | 0.740 | 1.35 | 2.67 | 0.626 | 1.66 | 2.73 | 0.559 |
|  | Urban | -0.56 | 1.18 | 0.646 | 2.21 | 1.49 | 0.176 | 2.77 | 1.96 | 0.194 |
| Manchester | Rural | -1.05 | 0.82 | 0.236 | -0.71 | 1.02 | 0.507 | 0.34 | 1.10 | 0.766 |
|  | Urban | -1.00 | 1.07 | 0.387 | 0.83 | 1.39 | 0.569 | 1.82 | 1.87 | 0.358 |
| Leeds | Rural | 0.25 | 0.98 | 0.805 | -3.50 | 3.13 | 0.295 | -3.75 | 3.26 | 0.282 |
|  | Urban | -1.01 | 0.99 | 0.335 | -0.83 | 2.39 | 0.739 | 0.19 | 2.59 | 0.945 |
| Newcastle | Rural | -0.05 | 1.42 | 0.973 | -1.44 | 4.17 | 0.738 | -1.39 | 3.62 | 0.711 |
|  | Urban | 1.08 | 1.18 | 0.389 | -1.18 | 2.84 | 0.690 | -2.25 | 2.48 | 0.391 |
| Glasgow | Rural | -0.52 | 0.55 | 0.376 | -1.78 | 1.15 | 0.160 | -1.26 | 1.16 | 0.308 |
|  | Urban | -0.32 | 1.02 | 0.760 | -1.41 | 1.12 | 0.243 | -1.09 | 1.43 | 0.468 |
| Edinburgh | Rural | -0.01 | 0.65 | 0.984 | 1.65 | 2.03 | 0.440 | 1.66 | 1.82 | 0.388 |
|  | Urban | -0.06 | 0.73 | 0.935 | 0.64 | 1.99 | 0.755 | 0.71 | 1.62 | 0.675 |
| All cities | Rural | -0.40 | 0.81 | 0.631 | -1.02 | 2.51 | 0.694 | -0.62 | 2.28 | 0.793 |
|  | Urban | -0.18 | 0.80 | 0.832 | -0.54 | 1.96 | 0.790 | -0.36 | 1.98 | 0.859 |

**Table S3:** Location and urban form characteristics Britain’s 15 largest cities (listed in order of ascending latitude).

| **City** | **Latitude** | **Longitude** | **Greenspace (%)^1^** | **Dwelling density (ha^-1^)^1^** | **Urban extent (ha)^1^** | **Distance to nearest major urban area (km)^2^** | **Disposable Household income in 2006(GB£)^3^** |
| --- | --- | --- | --- | --- | --- | --- | --- |
| Bournemouth | 50.72 | -1.88 | 0.24 | 13.90 | 7435.17 | 60 | 14840 |
| Portsmouth | 50.77 | -1.08 | 0.21 | 25.68 | 4418.88 | 60 | 10768 |
| Brighton | 50.83 | -0.15 | 0.17 | 38.76 | 3463.86 | 70 | 14995 |
| Bristol | 51.45 | -2.58 | 0.18 | 19.01 | 8632.02 | 100 | 12786 |
| London | 51.50 | -0.12 | 0.28 | 23.34 | 126097.17 | 80 | 18319 |
| Birmingham | 52.47 | -1.92 | 0.21 | 15.89 | 58090.80 | 50 | 11706 |
| Leicester | 52.63 | -1.13 | 0.20 | 18.41 | 7324.65 | 40 | 10336 |
| Nottingham | 52.97 | -1.17 | 0.19 | 21.28 | 7736.40 | 40 | 9861 |
| Sheffield | 53.37 | -1.50 | 0.23 | 17.68 | 11669.70 | 50 | 12002 |
| Liverpool | 53.42 | -3.00 | 0.26 | 20.35 | 11928.42 | 50 | 11251 |
| Manchester | 53.50 | -2.22 | 0.29 | 18.32 | 49221.06 | 50 | 12280 |
| Leeds | 53.80 | -1.58 | 0.40 | 19.80 | 14107.65 | 50 | 12127 |
| Newcastle | 54.99 | -1.62 | 0.28 | 19.41 | 17233.80 | 130 | 11638 |
| Glasgow | 55.83 | -4.25 | 0.36 | 15.80 | 13773.33 | 70 | 12908 |
| Edinburgh | 55.95 | -3.20 | 0.40 | 28.38 | 9761.82 | 70 | 15770 |

1 source: Dallimer et al (2011)

2 measured in a GIS between city centre to city centre and rounded to the nearest 10km

3 source: ONS (2012)

**Table S4:**

For each individual city (listed in order of ascending latitude), vegetation growing season median start and end Julian days, and length of growing season (SOS, EOS and LOS respectively). Median of differences, as displayed in Figure 3, between urban and rural zones (i.e. the median of the annual differences), with significance (p<0.05; Wilcoxon signed-rank tests) highlighted in bold.

| **City** | **Zone** | **SOS** | | | **EOS** | | | **LOS** | | |
| --- | --- | --- | --- | --- | --- | --- | --- | --- | --- | --- |
|  |  | **Median** | **V** | **P** | **Median** | **V** | **p** | **Median** | **V** | **P** |
| Bournemouth | Urban | 101.78 |  |  | 302.16 |  |  | 207.03 |  |  |
|  | Rural | 94.72 |  |  | 300.14 |  |  | 200.88 | 24 | 0.770 |
|  | Median of differences | ***4.11*** | ***4*** | ***0.014*** | 1.72 | 14 | 0.193 | -3.30 | 24 | 0.770 |
| Portsmouth | Urban | 89.35 |  |  | 306.75 |  |  | 221.55 |  |  |
|  | Rural | 94.40 |  |  | 297.19 |  |  | 205.41 |  |  |
|  | Median of differences | -4.41 | 43 | 0.131 | 1.65 | 17 | 0.322 | 11.29 | 9 | 0.065 |
| Brighton | Urban | 88.92 |  |  | 279.53 |  |  | 189.26 |  |  |
|  | Rural | 85.58 |  |  | 276.26 |  |  | 189.56 |  |  |
|  | Median of differences | 0.82 | 17 | 0.322 | 3.27 | 17 | 0.322 | 3.26 | 37 | 0.375 |
| Bristol | Urban | 95.06 |  |  | 288.85 |  |  | 197.04 |  |  |
|  | Rural | 88.24 |  |  | 275.92 |  |  | 188.83 |  |  |
|  | Median of differences | 7.01 | 10 | 0.084 | ***10.59*** | ***8*** | ***0.049*** | 6.60 | 19 | 0.432 |
| London | Urban | 91.42 |  |  | 287.72 |  |  | 193.97 |  |  |
|  | Rural | 90.50 |  |  | 284.25 |  |  | 191.24 |  |  |
|  | Median of differences | -1.58 | 43 | 0.131 | ***3.50*** | ***8*** | ***0.049*** | 7.10 | 9 | 0.065 |
| Birmingham | Urban | 100.26 |  |  | 278.76 |  |  | 178.79 |  |  |
|  | Rural | 98.85 |  |  | 268.24 |  |  | 182.06 |  |  |
|  | Median of differences | 2.46 | 13 | 0.160 | 2.82 | 17 | 0.322 | 0.25 | 28 | 0.995 |
| Leicester | Urban | 86.02 |  |  | 282.92 |  |  | 195.30 |  |  |
|  | Rural | 81.60 |  |  | 246.74 |  |  | 161.33 |  |  |
|  | Median of differences | -0.24 | 21 | 0.557 | 12.46 | 11 | 0.106 | 13.06 | 10 | 0.084 |
| Nottingham | Urban | 96.43 |  |  | 284.58 |  |  | 190.19 |  |  |
|  | Rural | 88.57 |  |  | 262.10 |  |  | 167.21 |  |  |
|  | Median of differences | -0.18 | 25 | 0.846 | 10.92 | 14 | 0.193 | 13.27 | 13 | 0.160 |
| Sheffield | Urban | 101.07 |  |  | 289.83 |  |  | 188.42 |  |  |
|  | Rural | 98.38 |  |  | 283.58 |  |  | 182.13 |  |  |
|  | Median of differences | -1.13 | 36 | 0.432 | 1.09 | 16 | 0.160 | ***2.22*** | ***6*** | ***0.027*** |
| Liverpool | Urban | 95.61 |  |  | 290.99 |  |  | 195.96 |  |  |
|  | Rural | 103.06 |  |  | 289.79 |  |  | 185.09 |  |  |
|  | Median of differences | ***-5.80*** | ***55*** | ***0.002*** | 2.10 | 13 | 0.160 | ***14.47*** | ***3*** | ***0.010*** |
| Manchester | Urban | 102.57 |  |  | 291.67 |  |  | 185.80 |  |  |
|  | Rural | 105.67 |  |  | 285.59 |  |  | 180.01 |  |  |
|  | Median of differences | ***-4.11*** | ***55*** | ***0.002*** | 0.90 | 19 | 0.432 | ***5.17*** | ***7*** | ***0.037*** |
| Leeds | Urban | 96.93 |  |  | 282.78 |  |  | 189.85 |  |  |
|  | Rural | 88.86 |  |  | 271.55 |  |  | 183.99 |  |  |
|  | Median of differences | ***3.08*** | ***3*** | ***0.010*** | ***2.96*** | ***7*** | ***0.037*** | -0.46 | 29 | 0.922 |
| Newcastle | Urban | 90.54 |  |  | 280.36 |  |  | 191.23 |  |  |
|  | Rural | 93.41 |  |  | 268.21 |  |  | 176.63 |  |  |
|  | Median of differences | -5.78 | 33 | 0.625 | 6.74 | 18 | 0.375 | 8.32 | 13 | 0.160 |
| Glasgow | Urban | 99.68 |  |  | 288.09 |  |  | 187.83 |  |  |
|  | Rural | 100.70 |  |  | 286.29 |  |  | 182.53 |  |  |
|  | Median of differences | -2.31 | 36 | 0.432 | ***1.99*** | ***5*** | ***0.020*** | ***5.71*** | ***5*** | ***0.019*** |
| Edinburgh | Urban | ***93.33*** |  |  | 281.85 |  |  | ***190.58*** |  |  |
|  | Rural | ***98.62*** |  |  | 279.08 |  |  | ***174.42*** |  |  |
|  | Median of differences | ***-5.63*** | ***48*** | ***0.037*** | 5.44 | 11 | 0.106 | ***10.56*** | ***0*** | ***0.002*** |

**References**

Dallimer M, Tang ZY, Bibby PR, Brindley P, Gaston KJ, Davies ZG (2011). Temporal changes in greenspace in a highly urbanized region. Biology Letters 7: 763-766.

ONS (2006) *UK’s major Urban Areas*. In Focus on people and migration, pp. 45-60. Newport: Office for National Statistics.

ONS (2012) *Household Disposable Income across the UK*. Office for National Statistics, London.
